# Death in Prison: increasing transparency on next of kin notification and disposition of remains

**DOI:** 10.1186/s40352-023-00232-x

**Published:** 2023-09-12

**Authors:** Yoshiko Iwai, Michael Forrest Behne, Lauren Brinkley-Rubinstein

**Affiliations:** 1 University of North Carolina at Chapel Hill School of Medicine, 340 MacNider Hall Campus, 333 South Columbia Street, Box 7240, Chapel Hill, NC 27599-7240 USA; 2 Gillings School of Global Public Health, University of North Carolina at Chapel Hill, Chapel Hill, NC USA; 3 Department of Population Health Sciences, Duke University, Durham, NC USA

**Keywords:** Prisons, Next-of-kin notification, Communication, Death, Prison policies

## Abstract

**Background:**

Policies for next-of-kin (NOK) notification and disposition of remains surrounding death are unclear across the United States’ (US) carceral systems. The goal of this study was to collect data on carceral system policies pertaining to NOK notification and disposition of remains for individuals who are incarcerated. We collected publicly available operational policies for the Federal Bureau of Prisons, Immigration and Customs Enforcement, 50 state prison systems, and the Washington D.C. jail for a total of 53 systems.

**Results:**

Approximately 70% of systems had available policies on NOK notification and disposition of remains. Few systems had information on time constraints for NOK notification, notifying parties or designated contacts person, and ultimate disposition of unclaimed remains. Several systems had no accessible policies.

**Conclusions:**

Across the US, carceral systems vary in policies for notifying NOK after the death of an incarcerated individual and their processes for the disposition of remains. Carceral and health systems should work towards standardization of policies on communication and disposition of remains after death of an individual who is incarcerated to work towards equity.

## Background

In the United States (US), approximately 600,000 people enter prisons each year (Sawyer & Wagner, 2022). Individuals who are incarcerated carry a high burden of severe and chronic disease including cancer, substance use disorders, and psychiatric conditions, which often remain undertreated (Logue et al., 2022; Oladeru et al., 2022; Wilper et al., 2009). Many challenges stand in the way of timely and complete healthcare for populations experiencing incarceration such as transportation, provider shortages, and stigma (Eisenstein et al., 2020; Puglisi & Wang, 2021). These challenges become pronounced in the process of death and dying among individuals who are incarcerated including challenges around obtaining palliative care (Linder & Meyers, 2007; Stephens et al., 2019).

Communicating the health status of individuals experiencing incarceration, including critical decisions at end of life, are often complicated and obscure (“How Should a Health Care Professional Respond to an Incarcerated Patient’s Request for a Particular Treatment?,” 2017; “Who Should Make Decisions for Unrepresented Patients Who Are Incarcerated?,” 2019). This is likely due to the complex interplay of structural barriers preventing individuals from exercising autonomy while incarcerated, lack of transparent policies, discrimination, and insufficient provider knowledge in advance care planning (Ekaireb et al., 2018; Frank et al., 2014; “Who Should Make Decisions for Unrepresented Patients Who Are Incarcerated?,” 2019). Another complicating factor may be related to the marked heterogeneity in state policies that can be appreciated in many areas of healthcare, including abortion, contraception, shackling, organ donation, and end-of-life care (Asiodu et al., 2021; Helmly et al., 2022; Iwai et al., 2023; Pan et al., 2021; Sufrin et al., 2021). Yet, it is unknown how carceral systems differ in their policies for notifying next of kin in the time surrounding death and the processes for determining the disposition of remains, especially in cases where next-of-kin (NOK) communication is inaccessible. Understanding the process that follows death for individuals who are incarcerated is critical for ensuring equity.

There is currently no central database for stakeholders to review or consult policies on NOK notification and disposition of remains. The goal of this study was to collect data on carceral system policies pertaining to NOK notification and disposition of remains for individuals who are incarcerated. Given the wide heterogeneity in state prison policies surrounding healthcare decisions reported by the literature, we hypothesized there would be significant heterogeneity across systems in the US with some states having no accessible policy on these issues.

## Methods

We collected publicly available operational policies for the Federal Bureau of Prisons (BOP), Immigration and Customs Enforcement (ICE), 50 state prison systems, and the Washington D.C. jail for a total of 53 systems. Policies were accessed on governmental websites that were publicly accessible. We used an inductive coding approach to analyze the policies for NOK notification and disposition of remains (Bradley et al., 2007). The inductive approach involved line-by-line review of all data elements until a concept emerged and code could be applied (Bradley et al., 2007). All policies were coded using a spreadsheet in an encrypted database (Microsoft Excel, 2019, Version 16, Redmond, WA: Microsoft Corporation). Coding was completed by one primary member of the research team and a second reviewer was consulted for ambiguous or conflicting policies. Data were summarized as tables.

The genericized “Department of Correction (DOC)” is used when referencing the state or federal government carceral systems. For example, the New York State Department of Corrections and Community Supervision (NYSDOCCS) was simplified to DOC. This study was reviewed by the University of North Carolina at Chapel Hill Institutional Review Board and determined to be non-human subject research.

## Results

Policies on NOK notification are summarized in Table 1. There were 35 systems (not including the federal BOP and ICE) with publicly accessible policies on NOK notification after death. Those 35 systems also had a policy mandating contact with NOK after death. Only 25 systems had specific policies on who should contact the NOK. The notifying party to NOK varied by system, and included: Warden (Delaware, Georgia, Indiana, Kansas, South Dakota), Chaplain (Arizona, Nevada, Rhode Island, South Carolina, Texas, Washington DC), Superintendent (Alaska, Massachusetts, New York), Facility head (Idaho, Oklahoma), or a combination of chaplains, administrative directors, and coroners. The time limit for contacting NOK was established in six states: Arizona (1 h within the decision to notify), Hawaii (48 h), Kansas (6 h), Nevada (72 h), South Dakota (24 h), and Washington DC (8 h).

**Table 1:** Carceral system policies on Next-of-Kin notification for the death of an individuals who are incarcerated

Jurisdiction	Policy on NOK Notification (Y/N)	Policy Mandate Contact with NOK (Y/N)	Specification for Who Contacts NOK (Y/N)	Notifying Party	Time Limit (Y/N)	Time Limit (hr)
Federal BOP	Y	Y	Y	Warden	N	NR
ICE	Y	Y	Y	Applicable consular officials	Y	24
Alabama	N	N	N	N	N	NR
Alaska	Y	Y	Y	Superintendent	N	NR
Arizona	Y	Y	Y	Chaplain	Y	1
Arkansas	N	N	N	N	N	N
California	Y	Y	N	NR	N	NR
Colorado	Y	Y	Y	Facility health administrator or coroner	N	NR
Connecticut	Y	Y	Y	Facility chaplain, unit administrator, or parole authority	N	NR
Delaware	Y	Y	Y	Warden	N	NR
Florida	Y	Y	N	NR	N	NR
Georgia	Y	Y	Y	Warden	N	NR
Hawaii	Y	Y	N	NR	Y	48
Idaho	Y	Y	Y	Facility Head	N	NR
Illinois	Y	Y	Y	Chief Administrator	N	NR
Indiana	Y	Y	Y	Warden	N	NR
Iowa	Y	Y	N	NR	N	NR
Kansas	Y	Y	Y	Warden	Y	6
Kentucky	Y	Y	N	NR	N	NR
Louisiana	N	N	N	N	N	N
Maine	Y	Y	Y	Unit Manager	N	NR
Maryland	N	N	N	NR	N	NR
Massachusetts	Y	Y	Y	Superintendent	N	NR
Michigan	Y	Y	N	NR	N	NR
Minnesota	Y	Y	Y	Facility religious coordinator	N	NR
Mississippi	N	N	N	N	N	N
Missouri	N	N	N	N	N	N
Montana	N	N	N	NR	N	NR
Nebraska	N	N	N	N	N	N
Nevada	Y	Y	Y	Chaplain	Y	72
New Hampshire	N	N	N	NR	N	NR
New Jersey	Y	Y	Y	Social Services Staff	N	NR
New Mexico	N	N	N	N	N	N
New York	Y	Y	Y	Superintendent or Officer of the Day	N	NR
North Carolina	Y	Y	N	NR	N	NR
North Dakota	N	N	N	NR	N	NR
Ohio	Y	Y	Y	Managing officer	N	NR
Oklahoma	Y	Y	Y	Facility Head	N	NR
Oregon	Y	Y	Y	“Facility Contact Person”	N	NR
Pennsylvania	Y	Y	Y	Facility Manager	N	NR
Puerto Rico	N	N	N	N	N	N
Rhode Island	Y	Y	Y	Chaplain	N	NR
South Carolina	Y	Y	Y	Chaplain	N	NR
South Dakota	Y	Y	Y	Warden	Y	24
Tennessee	N	N	N	NR	N	NR
Texas	Y	Y	Y	Chaplain	N	NR
Utah	Y	Y	N	NR	N	NR
Vermont	Y	Y	N	NR	N	NR
Virginia	N	N	N	NR	N	NR
Washington*	N	N	N	NR	N	NR
Washington, D.C.	Y	Y	Y	Chaplain; Metropolitan Police Dept. (for community supervision deaths)	Y	8
West Virginia	N	N	N	N	N	N
Wisconsin	Y	Y	N	NR	N	NR
Wyoming	N	N	N	N	N	N

Abbreviations: Federal BOP (Bureau of Prisons), ICE (Immigration and Customs Enforcement), NOK (next of kin), NR (not reported)

*Washington does maintain policies that establish next-of-kin as decision-makers in the disposition of remains, which implicitly includes an notification of death

The federal BOP and ICE had policies for NOK notification, mandating contact to NOK after death and an assigned notifying party (Table 1). Federal BOP indicated that the warden was the notifying party and ICE stated “applicable consular officials.” The time limit for contacting NOK for ICE was indicated as 24 h, while federal BOP had no indication.

Policies on disposition of remains are summarized in Table 2. There were 35 systems (not including the federal BOP and ICE) with publicly accessible policies on the disposition of remains. In 31 systems, the NOK had the right to claim remains. The coordinator for disposition of remains with NOK varied by system (Table 2). Policy regarding the amount of time to claim remains were available in 11 systems. Florida reported the amount of time to claim remains, vaguely, as a “medically acceptable period.” There were 12 states (not shown in table) that indicated the prison system had a relationship with a funeral home, to which remains are sent. These were Arizona, California, Colorado, Connecticut, Kansas, Massachusetts, Michigan, New Jersey, Oklahoma, Pennsylvania, South Carolina, and Texas. There were four systems that indicated having some relationship with a hospital or university for handling or receiving the remains of incarcerated individuals: Florida, Nevada, Texas, Utah. There were 27 systems with policies regarding unclaimed remains. If unclaimed, remains were indicated as being sent to various places depending on the state. Most systems indicated unclaimed remains as disposed of by the state DOC and some would be sent to a coroner, medical examiner, funeral home, or other program (e.g., Faith and Citizens Program Office in the state of Colorado).

**Table 2:** Carceral system policies on disposition of remains for individuals who are incarcerated

Jurisdiction	Disposition of Remains (Y/N)	NOK Right to Claim (Y/NR)	NOK Coordinator	Time to Claim Remains (days)	Policy on Unclaimed Remains (Y/N)	If Unclaimed
Federal BOP	Y	NR	Case Management Coordinator	NR	N	NR
ICE	Y	Y	NR	7	Y	ICE/ERO
Alabama	N	N	N	N	N	N
Alaska	Y	Y	ME	NR	Y	NR
Arizona	Y	Y	Warden	NR	Y	Contract mortuary
Arkansas	Y	NR	NR	NR	N	NR
California	Y	Y	NR	10	Y	DOC
Colorado	Y	Y	Facility health services administrator	180	Y	Faith and Citizens Programs office
Connecticut	Y	Y	Chaplain, unit administrator or Director	NR	Y	Designated funeral director
Delaware	N	N	N	N	N	N
Florida	Y	Y	NR	“medically acceptable period”	Y	Anatomical Board of UF
Georgia	Y	NR	Warden	NR	N	NR
Hawaii	Y	NR	NR	NR	N	NR
Idaho	Y	Y	Facility head	180	Y	DOC
Illinois	Y	NR	NR	NR	N	NR
Indiana	N	N	N	N	N	N
Iowa	Y	Y	NR	NR	N	NR
Kansas	Y	Y	Warden/Superintendent	4	Y	Secretary of corrections
Kentucky	N	N	N	N	N	N
Louisiana	N	N	N	N	N	N
Maine	Y	Y	Unit Manager	NR	Y	Chief Administrative Officer
Maryland	N	N	N	N	N	N
Massachusetts	Y	Y	Superintendent or ME	NR	Y	DOC
Michigan	Y	Y	NR	NR	Y	DOC
Minnesota	Y	Y	Warden’s office	NR	Y	DOC
Mississippi	N	N	N	N	N	N
Missouri	N	N	N	N	N	N
Montana	N	N	N	N	N	N
Nebraska	N	N	N	N	N	N
Nevada	Y	Y	NR	NR	Y	DOC
New Hampshire	N	N	N	N	N	N
New Jersey	Y	Y	Social Work Supervisor	NR	Y	ME, funeral home
New Mexico	N	N	N	N	N	N
New York	Y	Y	Superintendent and Chaplain	2	Y	DOC
North Carolina	Y	Y	Facility Head	10	Y	Office of the Chief Medical Examiner
North Dakota	Y	Y	Warden or Chaplain	NR	N	NR
Ohio	Y	Y	Managing officer	NR	Y	DOC
Oklahoma	Y	Y	NR	30	Y	Host facility; JBCC Cemetery
Oregon	Y	Y	NR	NR	Y	DOC
Pennsylvania	Y	Y	Chaplain	5	Y	Humanity Gifts Registry or DOC
Puerto Rico	N	N	N	N	N	N
Rhode Island	Y	Y	Office of Rehabilitative Services	30	Y	DOC
South Carolina	Y	Y	Chaplain	NR	N	NR
South Dakota	Y	Y	NR	2	Y	DOC
Tennessee	N	N	N	N	N	N
Texas	Y	Y	Chaplain	NR	Y	Huntsville Unit Warden (TDCJ cemetery)
Utah	Y	Y	Warden	NR	Y	ME
Vermont	Y	Y	Superintendent	NR	Y	DOC
Virginia	N	N	N	N	N	N
Washington	Y	Y	Coroner/ME or DOC employee	NR	Y	Coroner/ME
Washington, D.C.	Y	Y	Dept. of Human Services, Burial Assistance Unit	NR	N	NR
West Virginia	N	N	N	N	N	N
Wisconsin	Y	Y	NR	NR	Y	DOC
Wyoming	N	N	N	N	N	N

Abbreviations: Federal BOP (Bureau of Prisons), ICE (Immigration and Customs Enforcement), ERO (Enforcement and Removal Operations), ME (medical examiner); OCME (Office of the Chief Medical Examiner), DOC (Department of Corrections), NR (not reported), UF (University of Florida), JBCC (Jackie Brannon Correctional Center), TDCJ (Texas Department of Criminal Justice)

The federal BOP had no accessible information on NOK right to claim remains, amount of time to claim remains, relationship with a funeral home, relationship with a hospital or university, policy for unclaimed remains, or where unclaimed remains were sent. ICE had policies available on disposition of remains and indicated NOK have the right to claim remains within a seven-day time frame. Unclaimed remains of people detained by ICE were sent to ICE and Enforcement and Removal Operations (ERO).

## Discussion

In this study, we examined publicly accessible policies on NOK notification and disposition of remains, including unclaimed remains, for individuals incarcerated in US carceral systems. Approximately 70% of systems had policies on NOK notification and disposition of remains. Only a small subset of systems had specific information available on time constraints for NOK notification, notifying parties or designated contact persons, and ultimate disposition of unclaimed remains. Several states (Alabama, Arkansas, Louisiana, Mississippi, Missouri, Nebraska, New Mexico, Puerto Rico, West Virginia, and Wyoming) had no accessible policies on any of these issues and these systems were concentrated in the South.

As we had hypothesized, there was significant heterogeneity among systems relevant to how they approached NOK notification and disposition of remains. Some systems relied heavily on the warden or prison officials to coordinate dispositions and communications, while others relied on chaplains and outside programs (e.g., affiliate church groups, local cemetery). In many systems, it was unclear which authority figure was the primary decision-maker regarding unclaimed remains.

The lack of accessible policies raises serious concern around decency, humanity, and transparency in communication surrounding the death of an individual during incarceration. In states where there are no written mandates, the extent and method of communication for family involvement remains unknown. These critical gaps in policy feed into the larger lack of transparency relevant to health and death data (Berk et al., 2021; Peterson & Brinkley-Rubinstein, 2021). While many systems release population-level mortality reports periodically, these are not standardized across systems and do not encapsulate the specificities of coordinating post-humous processes. Because real-time data of this kind does not exist to date, there is no way to meaningfully confirm how deaths are communicated and ultimately handled, especially in the case of unclaimed remains and their disposition.

Ensuring appropriate NOK involvement in death and disposition of remains requires a systemic change towards the decarceralization of policies that exist in prison states (American Public Health Association, 2020). Explicitly, NOK regulations should match that of the general population and be mandated across systems with clearly defined chains of communication for cases where NOK cannot be immediately reached. More proximally, our findings reveal the need for the establishment of state and federal laws surrounding death in prison, the standardization of laws across states, and increased public accessibility of these guiding structures. An indeterminate and obscured death is not a contingency of incarceration. As policies and laws are developed in carceral and healthcare systems, it will be essential to integrate the input of people who are incarcerated to work towards equity (Berk et al., 2021). These laws should be used to inform local hospital policies and health professional education to ensure care teams can advocate for the patient throughout the process.

## Conclusion

Across the US, prisons vary in policies for notifying NOK after the death of an individual who is incarcerated and their processes for the disposition of remains. While many systems had some policy in place for NOK notification and disposition of remains, several systems had no policies available on any of these issues. The lack of transparency in these policies raises serious concern for how death is managed in prisons and the rate of adherence to such policies. Carceral systems should work towards standardization of policies and establishment of laws on NOK involvement, transparent communication, and disposition of remains after the death of an individual who is incarcerated.

## Data Availability

The datasets analysed during the current study are available from the corresponding author on reasonable request.
